# High-Intensity, Unilateral Resistance Training of a Non-Paretic Muscle Group Increases Active Range of Motion in a Severely Paretic Upper Extremity Muscle Group after Stroke

**DOI:** 10.3389/fneur.2015.00119

**Published:** 2015-05-27

**Authors:** M. A. Urbin, Michelle L. Harris-Love, Alex R. Carter, Catherine E. Lang

**Affiliations:** ^1^Program in Physical Therapy, Washington University School of Medicine, St. Louis, MO, USA; ^2^Georgetown University Medical Center, MedStar National Rehabilitation Hospital, Washington, DC, USA; ^3^Department of Neurology, Washington University School of Medicine, St. Louis, MO, USA; ^4^Program in Occupational Therapy, Washington University School of Medicine, St. Louis, MO, USA

**Keywords:** rehabilitation, stroke, cross education, resistance training, upper extremity, electrophysiology

## Abstract

Limited rehabilitation strategies are available for movement restoration when paresis is too severe following stroke. Previous research has shown that high-intensity resistance training of one muscle group enhances strength of the homologous, contralateral muscle group in neurologically intact adults. How this “cross education” phenomenon might be exploited to moderate severe weakness in an upper extremity muscle group after stroke is not well understood. The primary aim of this study was to examine adaptations in force-generating capacity of severely paretic wrist extensors resulting from high intensity, dynamic contractions of the non-paretic wrist extensors. A secondary, exploratory aim was to probe neural adaptations in a subset of participants from each sample using a single-pulse, transcranial magnetic stimulation (TMS) protocol. Separate samples of neurologically intact controls (*n* = 7) and individuals ≥4 months post stroke (*n* = 6) underwent 16 sessions of training. Following training, one-repetition maximum of the untrained wrist extensors in the control group and active range of motion of the untrained, paretic wrist extensors in the stroke group were significantly increased. No changes in corticospinal excitability, intracortical inhibition, or interhemispheric inhibition were observed in control participants. Both stroke participants who underwent TMS testing, however, exhibited increased voluntary muscle activation following the intervention. In addition, motor-evoked potentials that were unobtainable prior to the intervention were readily elicited afterwards in a stroke participant. Results of this study demonstrate that high-intensity resistance training of a non-paretic upper extremity muscle group can enhance voluntary muscle activation and force-generating capacity of a severely paretic muscle group after stroke. There is also preliminary evidence that corticospinal adaptations may accompany these gains.

## Introduction

Fatiguing muscle contractions reduce neural output from corticomotor regions (1–3). This compromises the ability to drive spinal motor neurons to threshold and activate skeletal muscle. Repeated bouts of high-intensity resistance training evoke lasting adaptations that function to maintain muscle activation and force output (4). These adaptations are not exclusive to the musculature targeted by training. In fact, unilateral resistance training at sufficiently high-intensity enhances force-generating capacity of the homologous, untrained musculature, a phenomenon known as the cross education effect (5–10).

The potential clinical application of the cross education effect in conditions characterized by unilateral motor deficits is gaining traction in biomedical science (11–13). Hemiparesis following stroke, for example, occurs in 80% of survivors (14, 15), making stroke a leading cause of disability in the western world (16, 17). To date, one study has examined the cross education phenomenon in persons with stroke (11). This study demonstrated that 6 weeks of training that involved intense isometric contractions of the non-paretic ankle dorsiflexors increased torque production and muscle activation of the paretic dorsiflexors. Thus, high intensity, unilateral resistance training may be an alternative for individuals who do not qualify for empirically supported motor retraining strategies [e.g., constraint-induced movement therapy (18), task-specific training (19), etc.] because the paretic musculature is initially too weak to train. Indeed, effective treatment alternatives are needed for individuals who cannot participate in retraining programs that leverage the nervous system’s adaptive capacity to reorganize following neurological damage (20).

Insufficient corticomotor output onto spinal motor neurons is a fundamental mechanism of paresis (21), which parallels the abovementioned effects of fatiguing muscle contractions in the neurologically intact motor system. A widely accepted theory of post-stroke behavioral dysfunction involves an imbalance in excitability between the primary motor cortex (M1) of the lesioned and non-lesioned cerebral hemispheres (22). Stated another way, excitability of the non-lesioned M1 increases, exerting an excessively inhibitory influence over the already less excitable lesioned M1 (23). Recent work has explored the neural adaptations mediating the cross education effect in healthy adults (24–27). One of the hypothesized adaptations that correlate with strength gains in the untrained muscle is a reduction in interhemispheric inhibition from the trained onto the untrained M1 (28). Thus, neural adaptations brought about by high-intensity, unilateral resistance training appear to be the inverse of the neurological dysfunction following stroke, inviting the possibility that it may be a viable intervention strategy in individuals with severe paresis.

If unilateral resistance training of the non-paretic musculature can restore sufficient movement production in the paretic musculature, then severely affected individuals could go on to participate in evidence-based therapies. The current study was an exploratory investigation of the cross education effect in stroke survivors with severe upper extremity paresis. A sample of neurologically intact adults of similar age underwent the same intervention to verify that the protocol elicited the effect in absence of stroke. It was hypothesized that both samples would exhibit the cross education effect.

## Materials and Methods

### Participants

Stroke participants were recruited from the Brain Recovery Core registry at Washington University School of Medicine. Online advertisements were posted to recruit potential control participants. Inclusion criteria for stroke participants were (1) clinical diagnosis of ischemic or hemorrhagic stroke as determined by a stroke neurologist; (2) ≥3 months post stroke; and (3) Medical Research Council Scale for Strength score of 0 (i.e., no movement) to 2 (i.e., movement with influence of gravity removed) in the paretic wrist extensors. Exclusion criteria for both control and stroke participants were (1) neurological conditions (other conditions for stroke participants); (2) presence of musculoskeletal conditions affecting the bones and/or soft tissues of the upper extremity; (3) history of resistance training involving the wrist extensors; (4) presence of aphasia; and (5) contraindications to transcranial magnetic stimulation (TMS) including history of seizure, prescribed medications that increase the risk of seizure, and/or presence of metal implants. All participants provided informed consent according to procedures established and approved by the Washington University Institutional Review Board and were compensated for their time.

### Experimental design

The current study was a prospective cohort, repeated-measures design, requiring a total of 8 weeks for each participant to complete. Baseline testing (pre-intervention^1^) took place immediately after the experimenter obtained informed consent and administered the Edinburgh Handedness Inventory (29). After a 4-week waiting period, participants returned to the laboratory for a second session of pre-intervention testing (pre-intervention^2^) (11, 30). Over the next 4 weeks, participants engaged in 16 sessions of progressive, unilateral resistance training (4 sessions/week × 4 weeks). Post-intervention testing took place within 1 week of the final training session. Previous studies investigating the cross education effect and associated neural adaptations have typically included a separate control group that does not undergo training (24–27). Participants in this current study served as their own controls from pre-intervention^1^ to pre-intervention^2^ testing to account for natural recovery and performance variability that may occur following stroke (31). The duration of the waiting period was set to match that of the training protocol to ensure that any natural recovery was from a period of equal duration.

### Strength training protocol

The dynamic contraction strength of the wrist extensor musculature was trained. There is evidence to suggest that training musculature of the dominant limb results in the greatest strength transfer (32), but stroke is not exclusive to a particular cerebral hemisphere. Approximately half of control participants, therefore, trained wrist extensors of the non-dominant limb. The non-paretic wrist extensors of stroke participants were trained. The wrist musculature was targeted because extending the wrist against gravity is functionally relevant in many goal-directed movements and is an important inclusion criterion for some motor retraining therapies following stroke (33).

**Figure 1:**
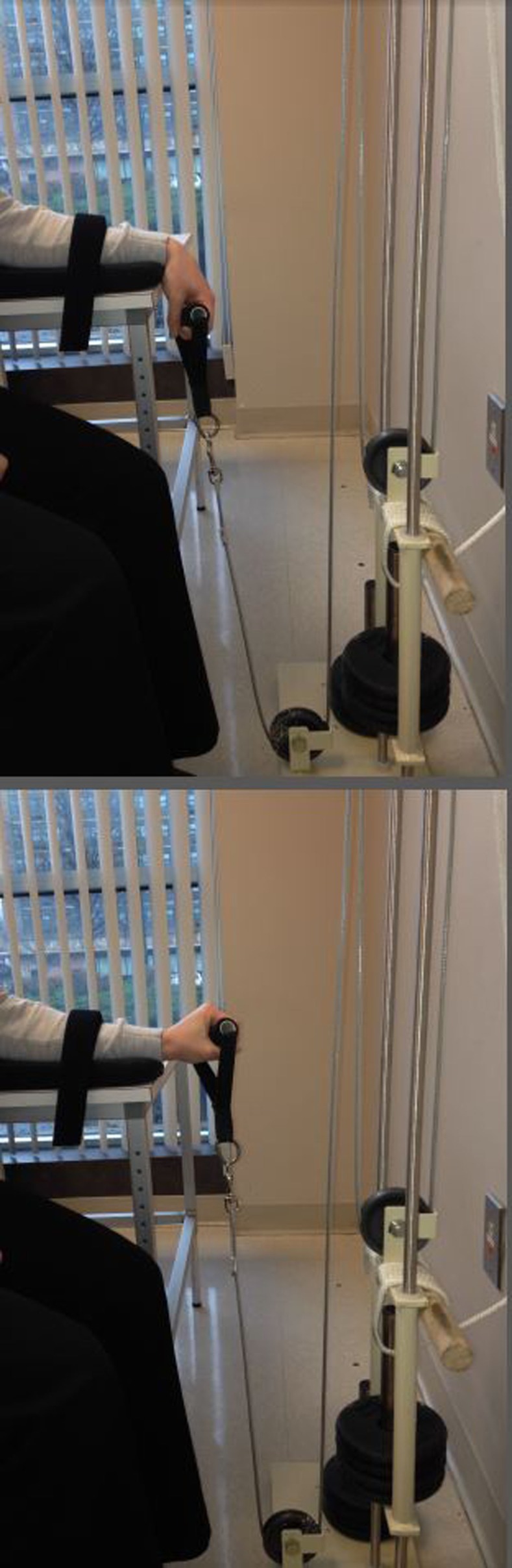
**Orientation of the hand relative to the forearm in the (top) starting position prior to each contraction and (bottom) position required for a repetition during training and 1-RM testing to be considered complete (i.e., dorsum of the hand extending above the plane parallel to the forearm)**.

A single-column pulley with stackable 0.5 kg barbells was used for training (Figure 1). Participants were seated in a chair with the shoulder abducted to ~45°. The forearm was pronated and strapped to a cushioned arm tray mounted to a table that was 23 cm away and 69 cm above the base of a pulley affixed to the resistance, which created a 72° angle from the pulley to the handle the participant grasped to perform wrist extensions. This orientation ensured that the resistance was applied throughout the entire range of motion. The resistance ascended the column during the concentric phase and descended during the eccentric phase. Once the experimenter brought the handle to the participant’s hand and verified that the wrist was fully flexed, the resistance was released and the participant initiated movement. Participants were verbally cued to perform the concentric and eccentric phases of the contraction at 3 and 4 s, respectively, as this timing has been reported to facilitate the greatest transfer of strength (5, 34). The forearm was held down to prevent the involvement/transfer of momentum from more proximal musculature. Prior to each training session, participants were guided through a warm-up (24, 26, 28). The warm-up consisted of 2 sets of continuous wrist oscillations for 30 s, 2, 10-s isometric contractions of the wrist extensors, and 2 cued sets of 6 repetitions on the trained movement at ~50% of one-repetition maximum (1-RM). 1-RM was evaluated during strength testing (see below) and is the maximum resistance the wrist extensors can contract against to produce an adequate range of motion for a repetition to be considered complete.

The initial volume (i.e., repetitions/set) and intensity (i.e., resistance) of training were selected based on a previous study that elicited the cross education effect and corticospinal adaptations projecting to an axial muscle of the upper extremity (25). Specifically, participants completed six sets with a goal of achieving six to eight repetitions during each set. Resistance was initially set at 80% of the participants’ 1-RM, as training intensity must be sufficiently high to elicit the effect (10, 35). Some participants in both groups could not achieve a minimum of six repetitions per set at this intensity during the initial training sessions. Resistance was, therefore, slightly reduced to ensure an adequate stimulus for maximizing strength gains. To minimize mirror movement, control participants were reminded to relax the untrained wrist and instructed to position the hand between the chair and thigh. Stroke participants attempted to do the same but were largely unable because mirror movements are exacerbated by hemiparetic stroke (36–38). Between each set, 90-s rest intervals were provided. Training was progressed (39) over the 16 sessions by increasing resistance in the subsequent session if 8 repetitions were achieved on 4 of the 6 sets (25). Thus, training load varied across participants and was contingent upon the ability to progress over the course of training. Training load was calculated by multiplying the total number of repetitions performed in each session by the respective percent of 1-RM training intensity, then summing the resulting value across all 16 sessions.

### Strength testing

In compliance with specificity principles of exercise testing and prescription, force-generating capacity of the trained, non-paretic wrist extensors was measured with the same single-column pulley used for training (40). The previously described warm-up protocol took place immediately before testing. Prior to 1-RM testing, the experimenter ensured that the participant’s feet were firmly positioned on the floor, the trunk was erect and positioned against the chair back, the handle was evenly and firmly grasped by the hand, and the participant indicated (s)he was prepared. The wrist was lowered into full flexion before the resistance was released and the participant was cued to begin the contraction. An attempt was considered as valid and complete if the dorsum of the hand extended above the plane parallel to the forearm (bottom image in Figure 1). In some cases, attempts that were near a resistance corresponding to a true 1-RM were on the border of this threshold, requiring another attempt to verify whether it was valid. Prior to each attempt, participants were reminded to contract with maximal effort and provided with verbal encouragement (3). Approximately 2–3 min of rest was allocated between attempts to minimize fatigue. The same procedures were administered to test the force-generating capacity of the untrained wrist extensors for control participants. The value for 1-RM at baseline and pre-intervention testing sessions was averaged before dividing by the value at post-intervention testing to calculate percent gain (11, 30).

Because the targeted subpopulation of stroke had severe paresis, paretic wrist extensor force-generating capacity was evaluated by measuring active range of motion (AROM) against gravity. AROM against gravity provides a measure of the ability to activate the target musculature when it is not strong enough to contract against resistance (41). Limb positioning for testing was identical to the abovementioned positioning for training. Initially, the paretic wrist was rotated through its full range of motion several times to warm-up the musculature and allow abnormal muscle tone to be evaluated via the modified Ashworth scale (42). The participant then performed wrist extensions until (s)he felt adequately prepared to start testing. The resting extension angle was measured from a fully relaxed position with a standard goniometer aligned parallel to the radial border of the second metacarpal with the capitate serving as the axis of rotation. Participants were then instructed to maximally extend the wrist against gravity. Cues such as “pull your hand back to your forearm” were provided until the wrist was maximally extended. The average of three separate attempts was recorded. Percent gain for AROM was calculated in the same way as 1-RM. For descriptive purposes, the action research arm test was also administered at pre-intervention^1^ to characterize movement capabilities of the paretic limb (43). This clinical scale measures the ability to reach, grasp, manipulate, and release objects. It consists of 19 items, each of which is scored on a 0–3 point scale, totaling 57 points, with higher scores indicating better movement capabilities.

### Neural adaptation testing

Transcranial magnetic stimulation was used to probe neural adaptations resulting from the intervention. Stimuli were delivered from a Magstim Rapid^2^ (Magstim Company Ltd., Wales, UK) with an encased figure-eight-shaped coil (70 cm diameter) oriented to elicit currents in a posterior–anterior direction. The extensor digitorum communis (EDC) was recorded from because it is involved in wrist extension and magnetic stimulation of its cortical representation has been reported to yield reliable responses (44). In addition, its distribution along the forearm is relatively narrow, constraining electrode placement to a more focal region thereby minimizing the potential for cross-talk in the electromyographic (EMG) signal, which is desirable given the difficulty of palpating a highly paretic muscle. A stereotactic neuronavigation system was used (Brainsight, Rogue Research, QC, Canada) to ensure the consistency of coil localization (i.e., position and orientation) across testing sessions. Prior to testing, skin overlying the EDC of both arms was abraded and cleansed with alcohol. Rectangular solid foam Ag–AgCl electrodes (7/8″ × 1.5″) with conductive gel were applied ~2 cm apart. To increase the reliability of electrode placement across testing sessions, the skin adjacent to the outermost corner of both electrodes was marked. Participants were encouraged to maintain and reinforce these marks during their participation. In anticipation of low adherence, photographs of electrode placement were taken at pre-intervention^1^. Amplified EMG signals were sampled at 3 kHz and recorded 100 and 600 ms pre- and post-stimulus, respectively, for off-line analysis.

The optimal scalp location for targeting the EDC was identified following behavioral testing at pre-intervention^1^. Stimulation of the lesioned hemisphere in stroke participants at the highest stimulator output did not produce motor-evoked potentials (MEPs) under resting conditions. Each stroke participant, therefore, was instructed to contract the paretic wrist extensors in an attempt to facilitate a response. If stimulation of the lesioned hemisphere could not produce MEPs, two steps were taken: (1) a stroke neurologist with training in TMS attempted to elicit MEPs independent of the primary experimenter, and (2) an approximate mirror location corresponding to the optimal cortical representation of the non-lesioned hemisphere was stimulated and saved for future testing. Recordings from stimulation at these sites were retained based on the assumption that if the intervention-induced neurological adaptations, then stimulating them at the post-intervention testing session may elicit MEPs (45, 46).

Prior to each testing session, resting motor threshold (RMT) was determined as the minimum stimulus intensity required to elicit MEPs of ≥50 μV in the relaxed EDC muscle on 5 of 10 consecutive trials (47). The M1 contralateral to the trained arm was probed to determine if the intervention altered corticospinal excitability and intracortical inhibition projecting to the trained musculature in control and stroke participants, which corresponded to the non-lesioned hemisphere and non-paretic arm of stroke participants. This was accomplished by measuring peak-to-peak MEP amplitudes at specific percentages of RMT and by quantifying the cortical silent period (CSP), respectively. MEP amplitude reflects the physiologic integrity of the corticospinal pathway (31), while the CSP is a measure of corticospinal inhibition (or disinhibition). Inhibitory mechanisms within the spinal cord are believed to contribute to the initial segment of the CSP (i.e., up to 50 ms) but the later segment (i.e., up to ~300 ms) is attributable to inhibition originating within M1 (48, 49), most likely due to the action of γ-aminobutyric acid (GABA) receptor-mediated interneuron transmission (50).

Ten individual stimuli were delivered to the trained/non-lesioned M1 at stimulator intensities corresponding to 90, 110, 130, and 150% of RMT with the targeted muscle at rest. Next, 10 individual stimuli were delivered at 150% of RMT with background activation of the trained/non-paretic EDC to elicit the CSP. The same protocol was used to evaluate corticospinal excitability in the untrained M1 of control participants. Absolute stimulator outputs were used for the untrained, lesioned M1 of stroke participants because MEPs could not be elicited at rest, which is common in individuals with severe paresis (51). The inability to elicit MEPs at rest, even with maximum stimulator output, prevented a RMT from being established. Ten stimuli, therefore, were delivered at 80, 90, and 100% of maximal stimulator output with maximal background activation of the untrained/paretic EDC.

Interhemispheric inhibition from the trained/non-lesioned M1 onto the untrained/lesioned M1 was evaluated by measuring the ipsilateral silent period (ISP). Twenty stimuli at 150% of RMT were delivered against background activation of the untrained/paretic EDC. Paired-pulse techniques commonly used to measure interhemispheric inhibition could not be administered because of the inability to reliably elicit MEPs from stimulation to the lesioned hemisphere of stroke participants. The ISP is a measure of interhemispheric inhibition and is thought to be mediated by excitatory projections from the stimulated M1 to inhibitory interneurons in the opposite M1 (52, 53). Absence of an ISP in individuals with agenesis of the corpus callosum (54) and in children before transcallosal fiber myelination (55) suggests that interhemispheric connections contribute to the ISP. Additional evidence comes from previous work showing that inhibition is preserved in patients with subcortical lesions that damage corticospinal fibers but spare transcallosal fibers (56). The same stimulation site was used to measure the ISP because there is correspondence in the cortical territory stimulated to elicit contralateral MEPs and ISPs (57, 58).

Timing of stimulus delivery (~0.1 Hz) for all conditions was randomized to prevent anticipation. Participants were trained to extend the wrist and middle digit to produce a stable, low-level contraction prior to conditions requiring background activation with a custom-made strain gage providing digital feedback. EMG recordings in the pre-stimulus window were inspected to verify that contraction magnitude and stability were similar across testing sessions. Due to paretic severity, stroke participants were instructed to maximally contract the wrist during active conditions. A brief period of rest (~10 s) occurred between the delivery of each stimulus to minimize the onset of fatigue and increased muscle tone.

### Data processing

Custom software was written in MATLAB (Mathworks, Inc., R2012a, Natick, MA, USA) to quantify electrophysiological parameters. Some participants in both samples were unable to maintain alertness and/or remain motionless during procedures involving TMS. Other participants exhibited large fluctuations in RMTs between the two pre-intervention test sessions. The inability to maintain stable arousal and remain still during stimulus delivery has been shown to increase the variability of EMG signals resulting from single-pulse TMS (59). Furthermore, large changes in RMT require concomitant changes in the stimulator output needed to measure intracortical and interhemispheric inhibition, which has a significant influence on silent periods (60, 61). Electrophysiological data, therefore, were analyzed from a subset of participants in each sample (control: *n* = 4, stroke: *n* = 2, asterisk in Table 1).

**Table 1:** **Demographic characteristics for stroke and control participants**.

Group	Age	Gender	Dominant side	Trained side	Race
**Control**					
C^1^*	31	Female	Right	Left	Caucasian
C^2^*	43	Female	Left	Right	Caucasian
C^3^	59	Female	Right	Left	African-American
C^4^*	57	Male	Right	Left	Caucasian
C^5^*	44	Female	Right	Right	Caucasian
C^6^	50	Male	Right	Right	Caucasian
C^7^	66	Female	Right	Left	Caucasian
**Stroke**					
S^1^	78	Male	Right	Left	Caucasian
S^2^	59	Male	Right	Right	African-American
S^3^	46	Male	Right	Right	African-American
S^4^	59	Male	Left	Left	Caucasian
S^5^*	38	Female	Right	Left	Caucasian
S^6^*	48	Female	Right	Left	Caucasian

*Participants tested for neural adaptations via TMS.

Samples with prolonged stimulus artifact were removed before averaging waveforms at each respective percentage of RMT. Peak-to-peak MEP amplitudes occurring between 10 and 55 ms post-stimulus were plotted against stimulation intensity to construct recruitment curves. The slope of the linear regression line was calculated using these values. For CSP measurements, individual trials were full-wave rectified before being averaged together. The mean pre-stimulus EMG amplitude from −100 to −10 ms (i.e., 10 ms before stimulus delivery) was calculated and used to set the threshold for silent period onset and offset. CSP onset was defined as the point at which the average rectified EMG amplitude remained below threshold for 5 ms; offset was defined as the point at which the amplitude returned to and remained above threshold for 5 ms. CSP duration was defined as the time between stimulus delivery and silent period offset (51). Samples at each percentage of stimulator output (i.e., 80, 90, and 100%) for the untrained/lesioned hemisphere of stroke participants were full-wave rectified before being averaged together. Area of the averaged waveform between 15 and 55 ms post-stimulus was calculated at each stimulator output. This area was normalized to the area of the pre-stimulus window.

For measurement of the ISP, individual trials were full-wave rectified before being averaged. Criteria for onset and offset were identical to those used for the CSP with the exception of the minimal latency (≥10 ms post-stimulus). The extent to which the signal amplitude was suppressed during the silent period was quantified by expressing the area between onset and offset as a percentage of an area of equal duration in the pre-stimulus window. The mean area of the pre-stimulus window (i.e., 90 ms) across all 20 trials used to measure the ISP was quantified to evaluate changes in muscle activation of stroke participants’ paretic wrist extensors.

### Statistical analysis

Shapiro–Wilk’s test and Mauchly’s test of sphericity indicated that data were normally distributed and variances were homogenous. Independent samples *t*-test were used to test for differences between groups in training load and percent gain in the trained and untrained wrist extensors. Repeated-measures analyses of variance (ANOVAs) were performed to determine the effect of time (pre-intervention^1^, pre-intervention^2^, post-intervention) and group (stroke, control) on 1-RM of the trained wrist extensors. Separate repeated-measures ANOVAs were used to determine the effect of time on 1-RM and AROM of the untrained wrist extensors for control and stroke groups, respectively. Pearson correlations were used to examine the relationship between training load and percent gain in the trained and untrained wrist extensors for both groups. All statistical procedures were performed using SPSS version 21 (IBM Statistics), and α level was set at 0.05 *a priori*. Since only a subset of stroke and control participants had viable electrophysiological data, these data were not subjected to formal statistical analysis.

## Results

Seven neurologically intact adults (five female; 50 ± 11.8 years of age) and six stroke survivors (two female; 54.7 ± 14.0 years of age) participated in the study. Demographics for both samples are shown in Table 1, and clinical characteristics of stroke participants are presented in Table 2. Stroke participants were between 4 months and ~2 years post stroke at the time they enrolled in the study with half affected on their dominant side. At pre-intervention^1^, four participants had a slight increase in wrist extensor muscle tone, and two participants had more pronounced muscle tone. All participants exhibited moderate-to-severe deficits in movement capabilities of the paretic limb, as reflected by total action research arm test score. Generally, grip and pinch scores were poorer than grasp and gross movement scores.

**Table 2:** **Clinical characteristics of stroke participants at pre-intervention^1^ testing**.

	Dominant side affected	Months post stroke	Stroke type	Stroke location	Modified ashworth	Action research arm test				
						Grasp	Grip	Pinch	Gross	Total
S^1^	Yes	20	Ischemic	Right MCA territory	1+	1	0	0	3	4
S^2^	No	13	Hemorrhagic	Left frontal	2	10	4	2	4	20
S^3^	No	7	Ischemic	Left medullary	1+	5	0	0	3	8
S^4^	No	7	Ischemic	Right MCA territory	3	0	0	0	3	3
S^5^	Yes	4	Ischemic	Subcortical white matter	1	4	2	0	4	10
S^6^	Yes	4	Ischemic	Right MCA territory	1	0	0	0	3	3

### Training load and strength gains of trained/non-paretic wrist extensors

**Figure 2:**
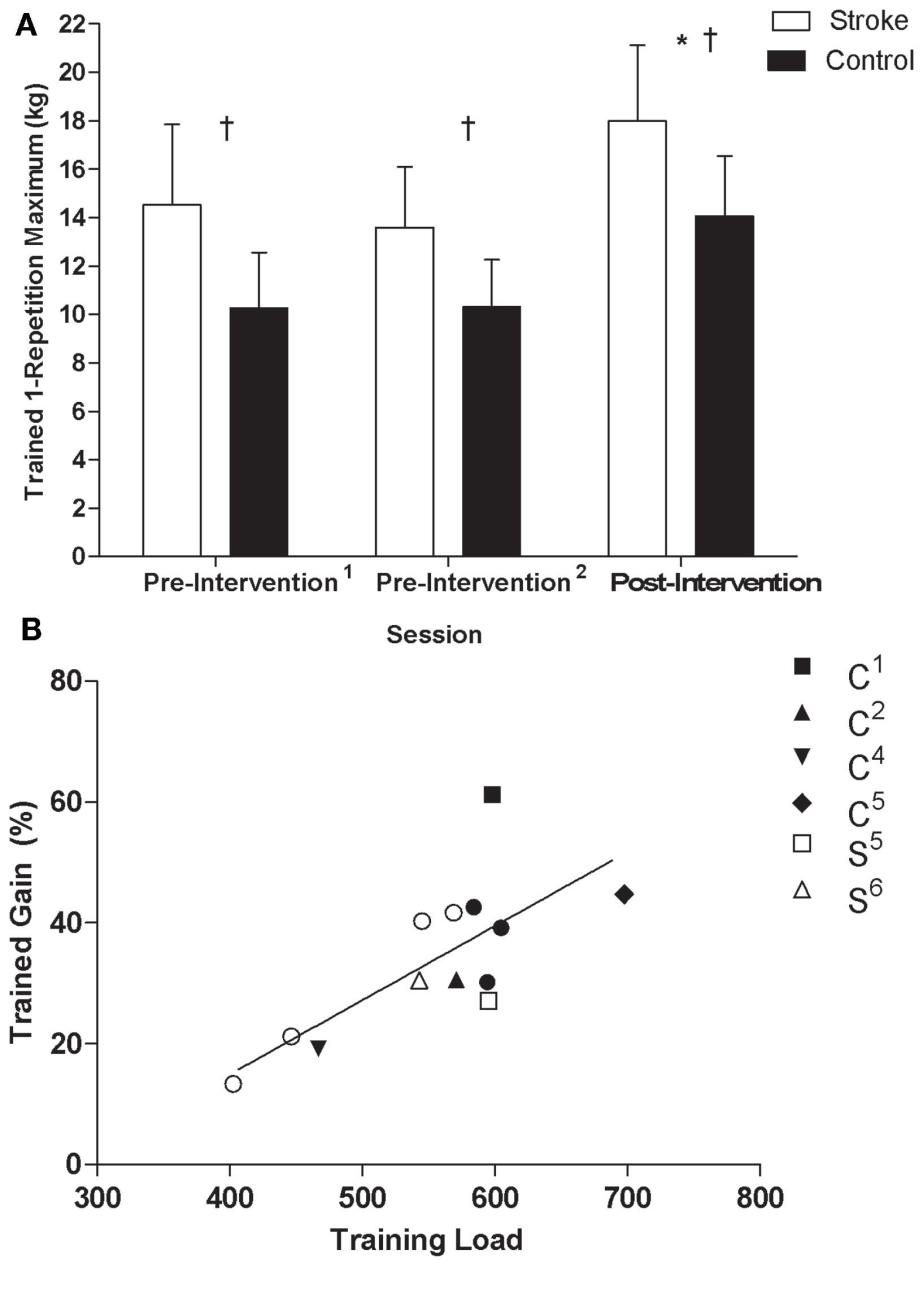
**(A)** 1-RM of the non-paretic/trained wrist extensors by testing session for control and stroke groups: *Significant difference (*p* < 0.05) relative to pre-intervention^1^ and pre-intervention^2^; ^†^significant difference (*p* < 0.05) between groups. **(B)** Scatterplots of training load and percent gain in the non-paretic/trained wrist extensors of stroke and control groups (*r* = 0.73, data pooled across groups, *p* < 0.05). Filled and unfilled symbols correspond to control and stroke participants, respectively. Unique symbols shown in the legend represent participants in either group that underwent TMS testing.

Training load (i.e., repetitions multiplied by percentage of 1-RM trained at for each session, summed across sessions) was not different between stroke and control participants (stroke = 517 ± 75, control = 588 ± 68, *t*_11_ = 1.8, *p* = 0.1), indicating that both groups were able to perform at a similar workload over the 16 training sessions. There was a significant effect of time (*F*_2,10_ = 58.73, *p* < 0.001, η^2^ = 0.92) and group (*F*_1,11_ = 7.36, *p* = 0.02, η^2^ = 0.40) on trained wrist extensor 1-RM but no interaction (*F*_2,22_ = 1.23, *p* = 0.31, η^2^ = 0.10, Figure 2A). *Post hoc* tests revealed that 1-RM was greater post-intervention than at both pre-intervention^1^ (*p* < 0.01) and pre-intervention^2^ (*p* < 0.01). Percent gain in 1-RM of the trained wrist extensors was not significantly different between groups (stroke = 29 ± 11%, control = 38 ± 13.4%, *p* = 0.2), indicating that the intervention evoked similar changes in strength of the trained musculature. There was a significant association between training load and percent gain in the trained wrist extensors (*r* = 0.73, data pooled across groups, *p* < 0.01, Figure 2B).

### Training load and strength gains of untrained/paretic wrist extensors

**Figure 3:**
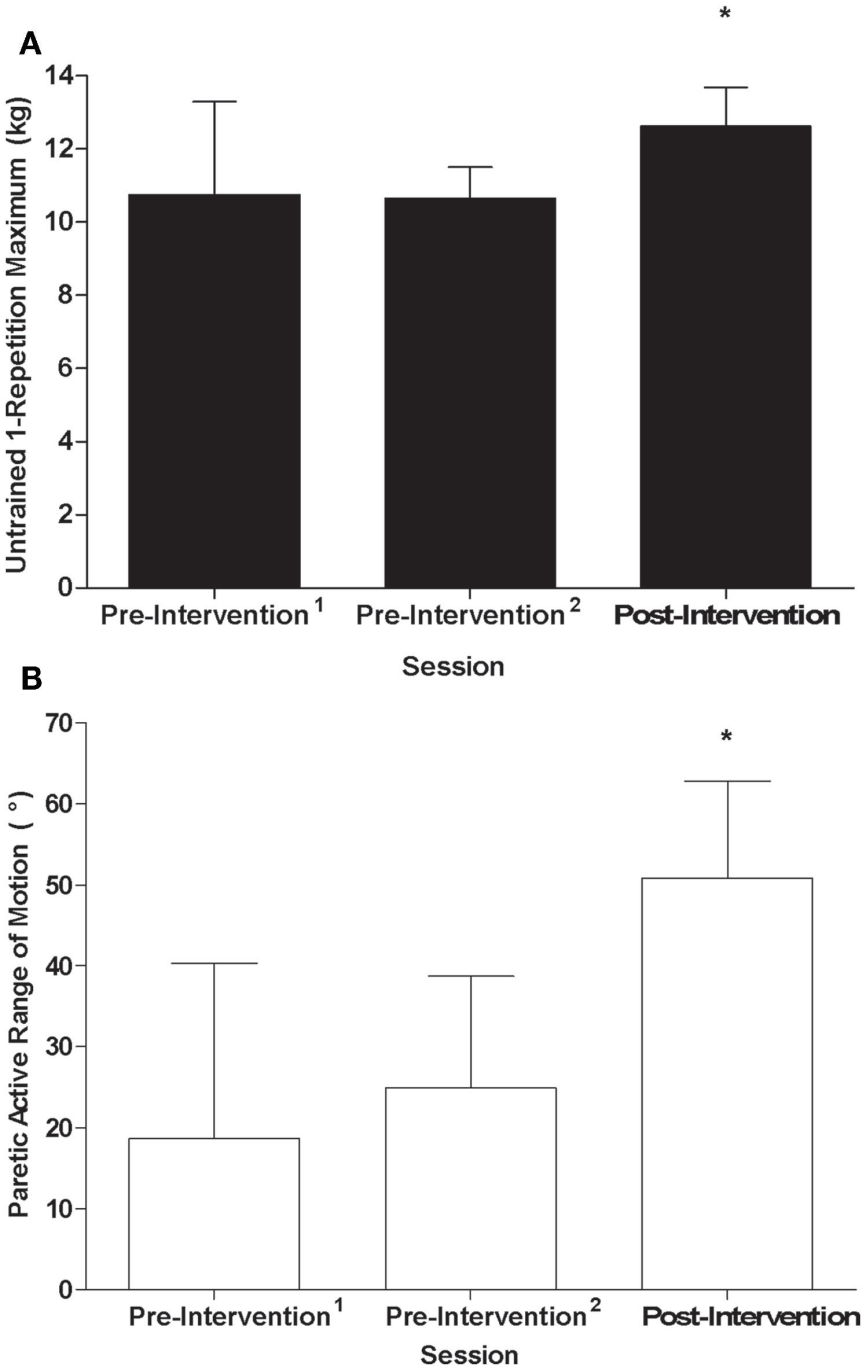
**(A)** 1-RM of the untrained wrist extensors by testing session for the control group; **(B)** AROM of the paretic, untrained wrist extensors by testing session for the stroke group: *Significant difference (*p* < 0.05) relative to pre-intervention^1^ and pre-intervention^2^.

**Figure 4:**
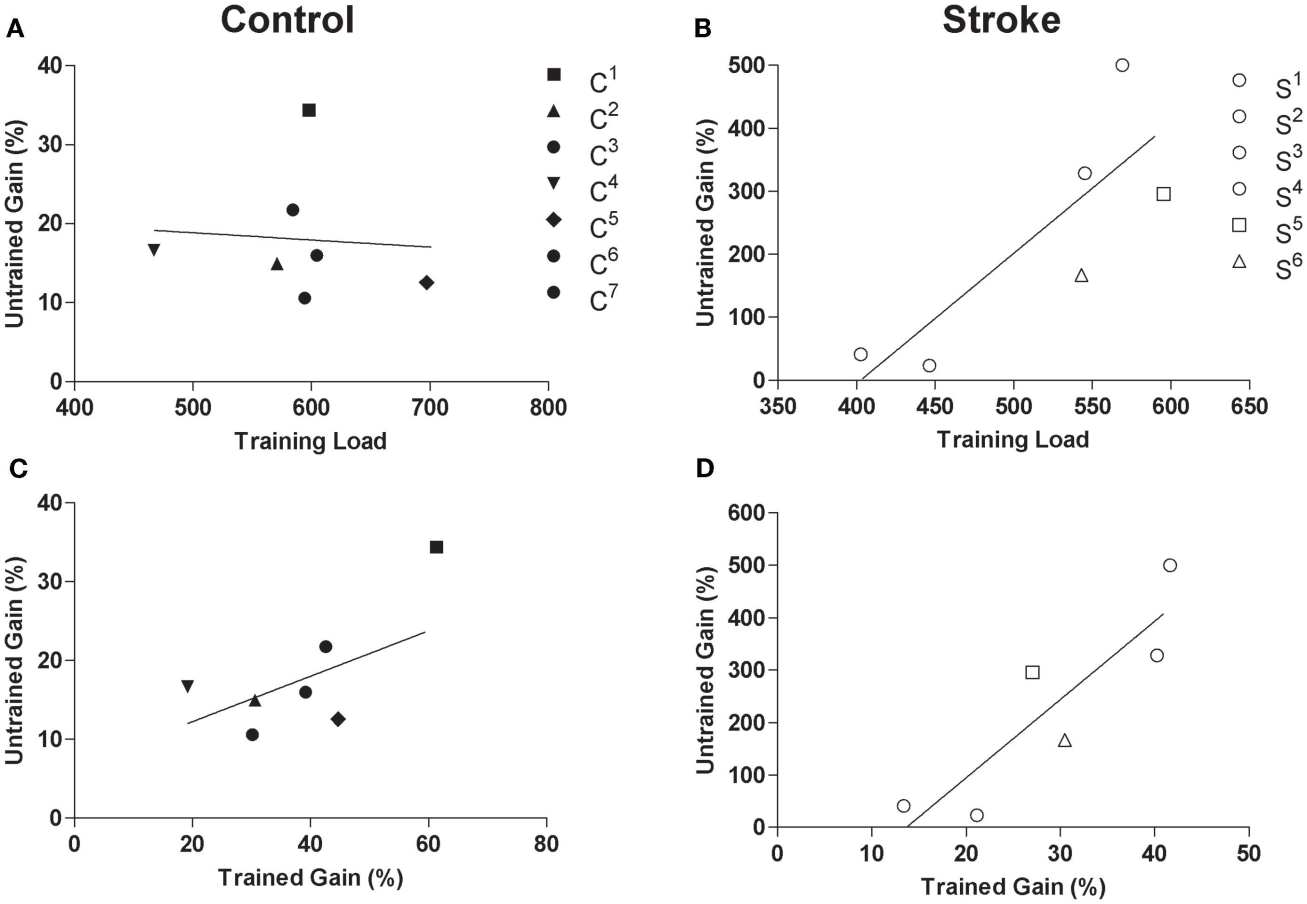
**Scatterplots of (A) training load and 1-RM of untrained wrist extensors for the control group (*r***=−**0.10, *p***=** 0.83), (B) training load and percent gain in AROM of paretic/untrained wrist extensors for stroke participants (*r***=** 0.82, *p***=** 0.05), (C) trained percent gain and untrained percent gain for control participants (*r***=** 0.73, *p***=** 0.07), and (D) non-paretic/trained percent gain and paretic/untrained percent gain for stroke participants (*r***=** 0.88, *p***=** 0.02)**. Filled and unfilled symbols correspond to control and stroke participants, respectively. Unique symbols shown in the legend represent participants in either group that underwent TMS testing.

A significant effect of time was observed for the 1-RM of untrained wrist extensors in the control group (*F*_2,5_ = 28.02, *p* < 0.01, η^2^ = 0.92, Figure 3A) where values were higher post-intervention compared to both pre-intervention^1^ and pre-intervention^2^ (*p* < 0.01). A significant effect of time was also present for AROM of the untrained, paretic wrist extensors in the stroke group (*F*_2,4_ = 15.63, *p* < 0.01, η^2^ = 0.89, Figure 3B), where AROM values were higher post-intervention relative to pre-intervention^1^ (*p* = 0.02) and pre-intervention^2^ (*p* < 0.01). Although there was no relationship between training load and percent gain of the untrained wrist extensors for controls (*r* = −0.10, *p* = 0.83, Figure 4A), there was an association between training load and percent gain of the paretic, untrained wrist extensors for the stroke group (*r* = 0.82, *p* = 0.05, Figure 4B). The association between percent gain of the untrained and trained wrist extensors was trending in the control group (*r* = 0.73, *p* = 0.07, Figure 4C) and was significant in the stroke group (*r* = 0.88, *p* = 0.02, Figure 4D). Total ARAT score showed a small but significant change from pre- to post-intervention: [$\bar{\text{x}}$pre = 7.8 ± 7.1, $\bar{\text{x}}$post = 10.2 ± 8.7, *t*(5) = −2.72, *p* = 0.04].

### Neural adaptations

**Figure 5:**
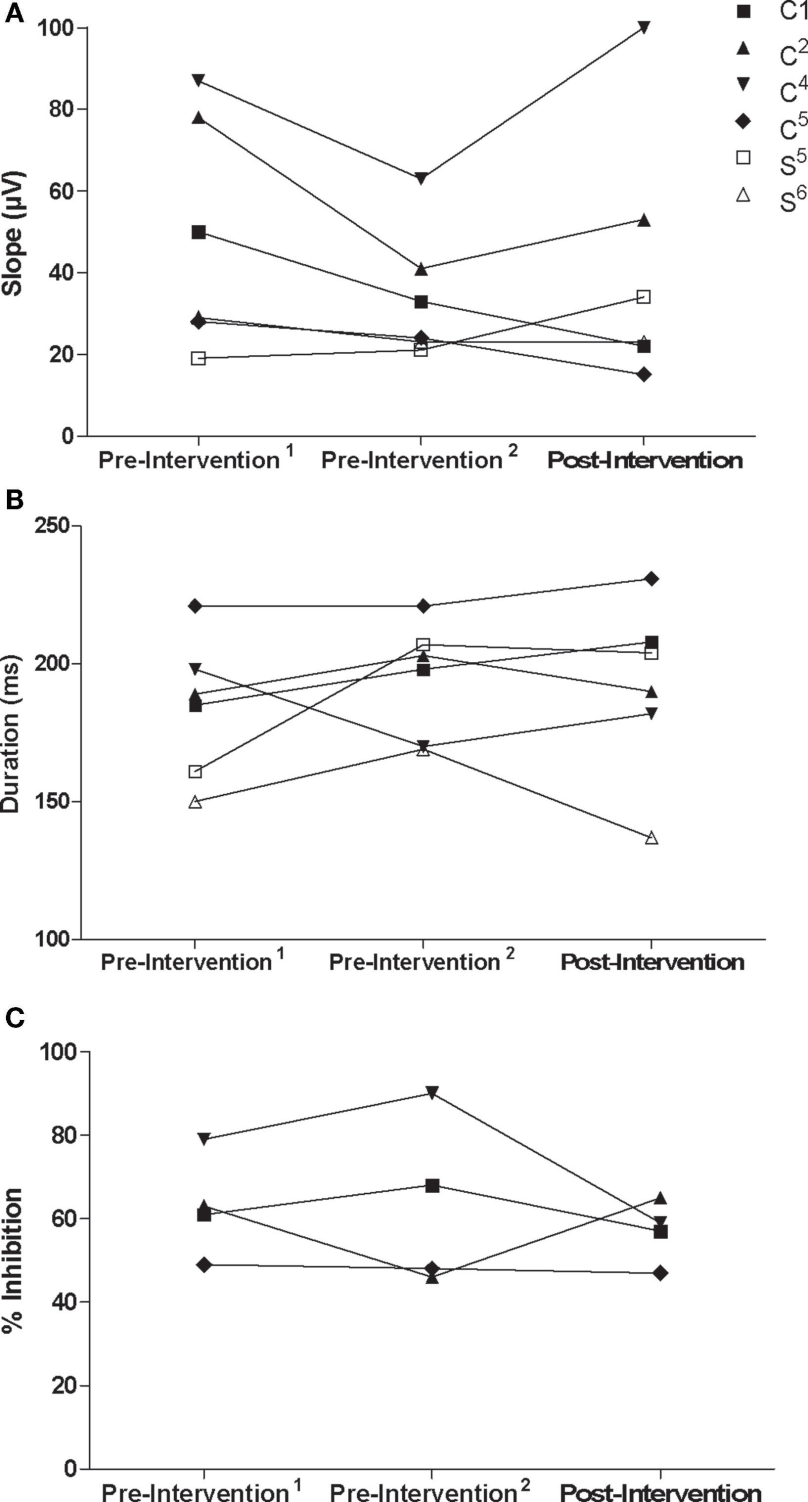
**(A)** Recruitment curve slope (microvolt) and **(B)** CSP (milliseconds) from the trained/non-lesioned M1 by testing session for each participant. **(C)** Ipsilateral silent period (% inhibition) from the trained M1 onto the untrained M1 by testing session for control participants.

**Figure 6:**
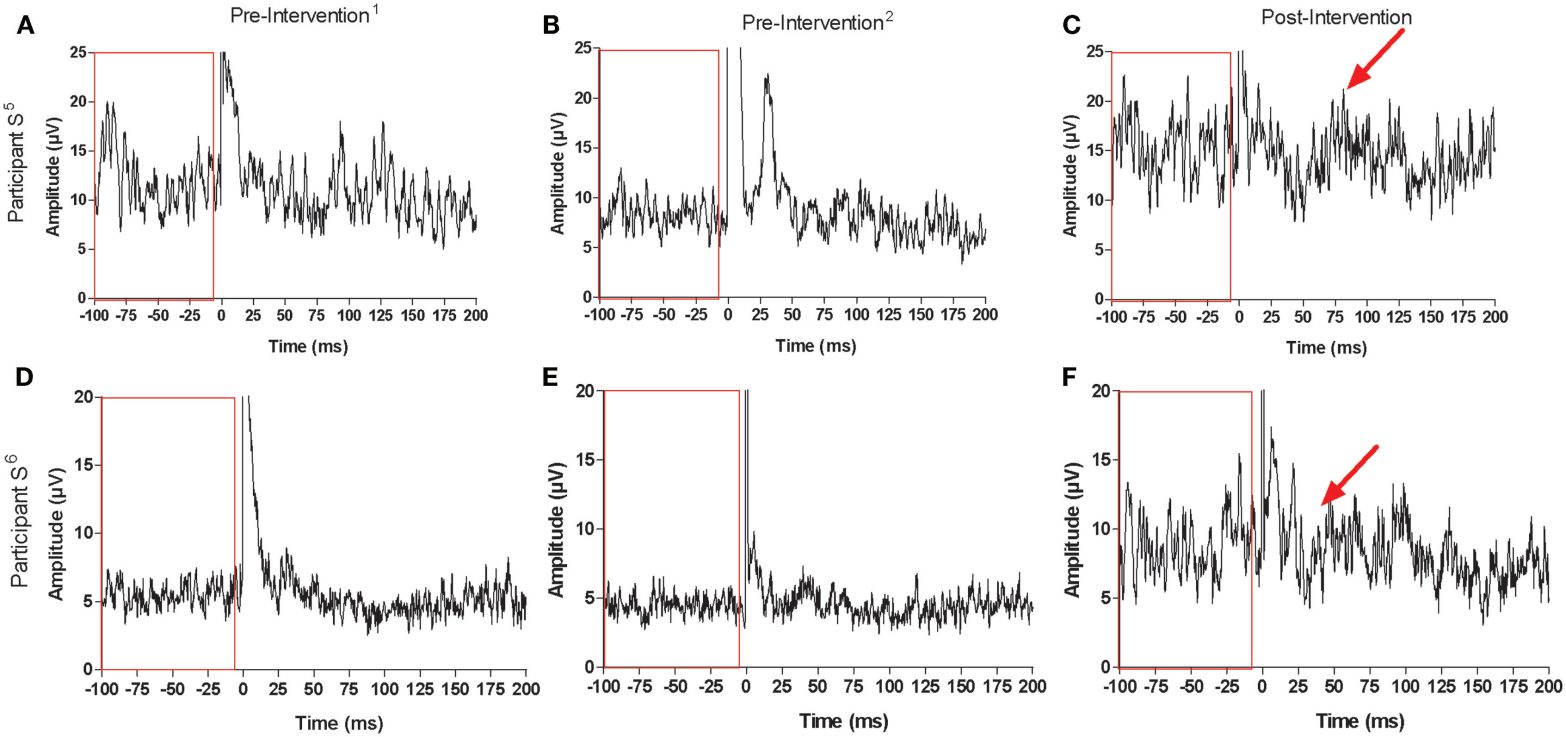
**Recordings for ISP and maximal background muscle activation at each testing session for stroke participant S^5^: [(A) pre-intervention^1^, (B) pre-intervention^2^, (C) post-intervention] and participant S^6^: [(D) pre-intervention^1^, (E) pre-intervention^2^, (F) post-intervention]**. Arrows point to suppression in the EMG signal that was not present before the intervention. Boxes contain pre-stimulus maximal muscle activation at each testing session, which increased for both stroke participants following the intervention (0 ms corresponds to stimulus delivery).

Recruitment curve slopes and CSP durations in the trained M1 were similar for control and stroke participants (Figures 5A,B). No consistent changes were observed in the trained M1 recruitment curve slope or CSP for either group. Across control participants, there was no consistent trend in interhemispheric inhibition from the trained M1 onto the untrained M1 across control participants (Figure 5C). There was no discernible ISP in either stroke participant at pre-intervention^1^ and pre-intervention^2^ testing sessions, which may have been attributable to an inability to activate the target muscle sufficiently. Ipsilateral MEPs were also observed in both participants at various testing sessions, which also made any suppression difficult to detect (Figures 6A–F). At post-intervention testing, however, there was noticeable suppression in the waveform at latencies similar to previous work involving individuals with more severe paresis following stroke (51). Nevertheless, the suppression was not sufficiently pronounced to quantify a silent period according to the pre-determined criteria. The area of pre-stimulus EMG was also greater at post-intervention testing (S^5^ = 4128 μV⋅ms; S^6^ = 2471 μV⋅ms) relative to both pre-intervention testing sessions (S^5^ = 2751 μV⋅ms; S^6^ = 1355 μV⋅ms) (Figures 6A–F).

**Figure 7:**
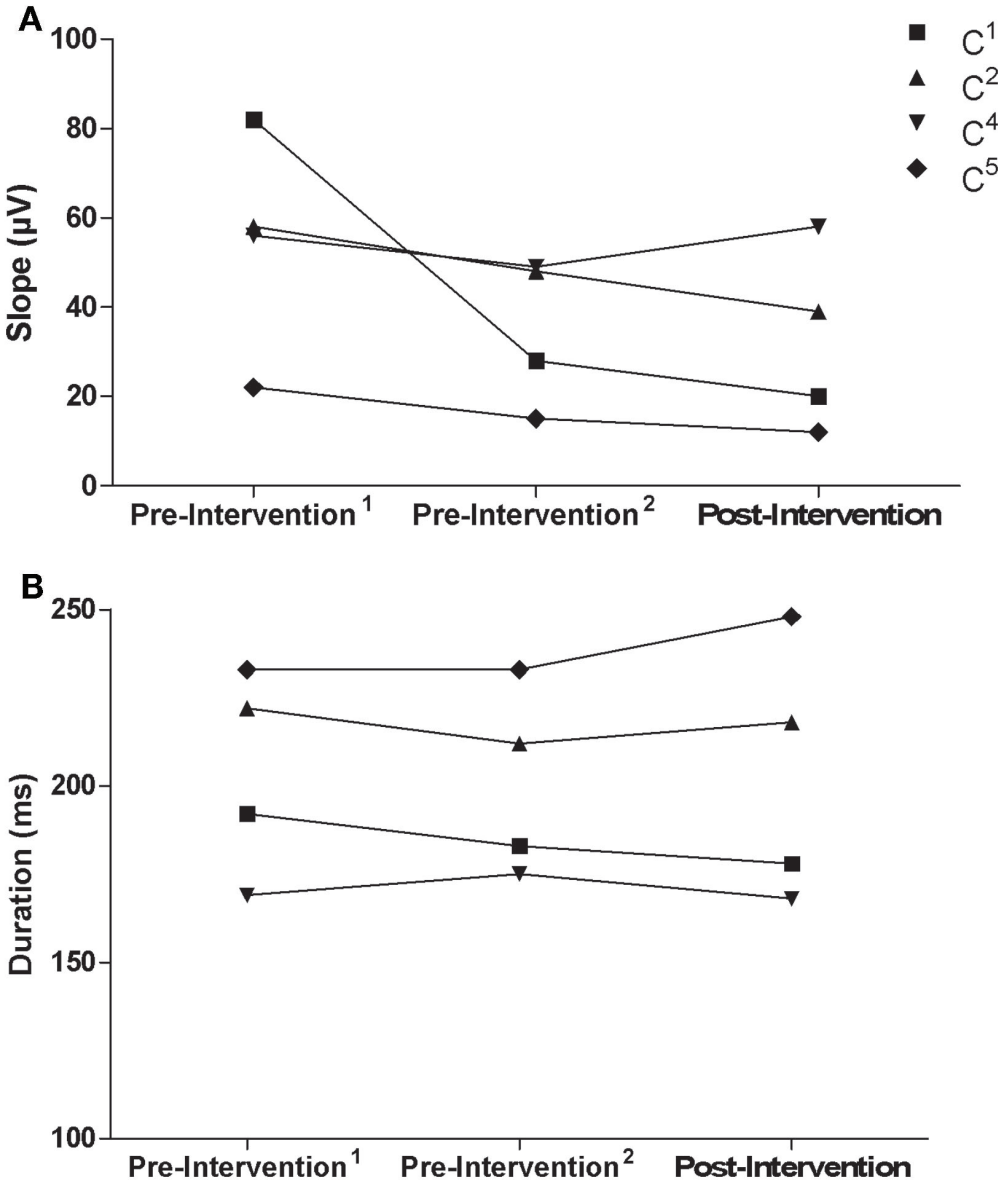
**(A)** Recruitment curve slope (microvolt) and **(B)** CSP (milliseconds) from the untrained M1 by testing session for control participants.

**Figure 8:**
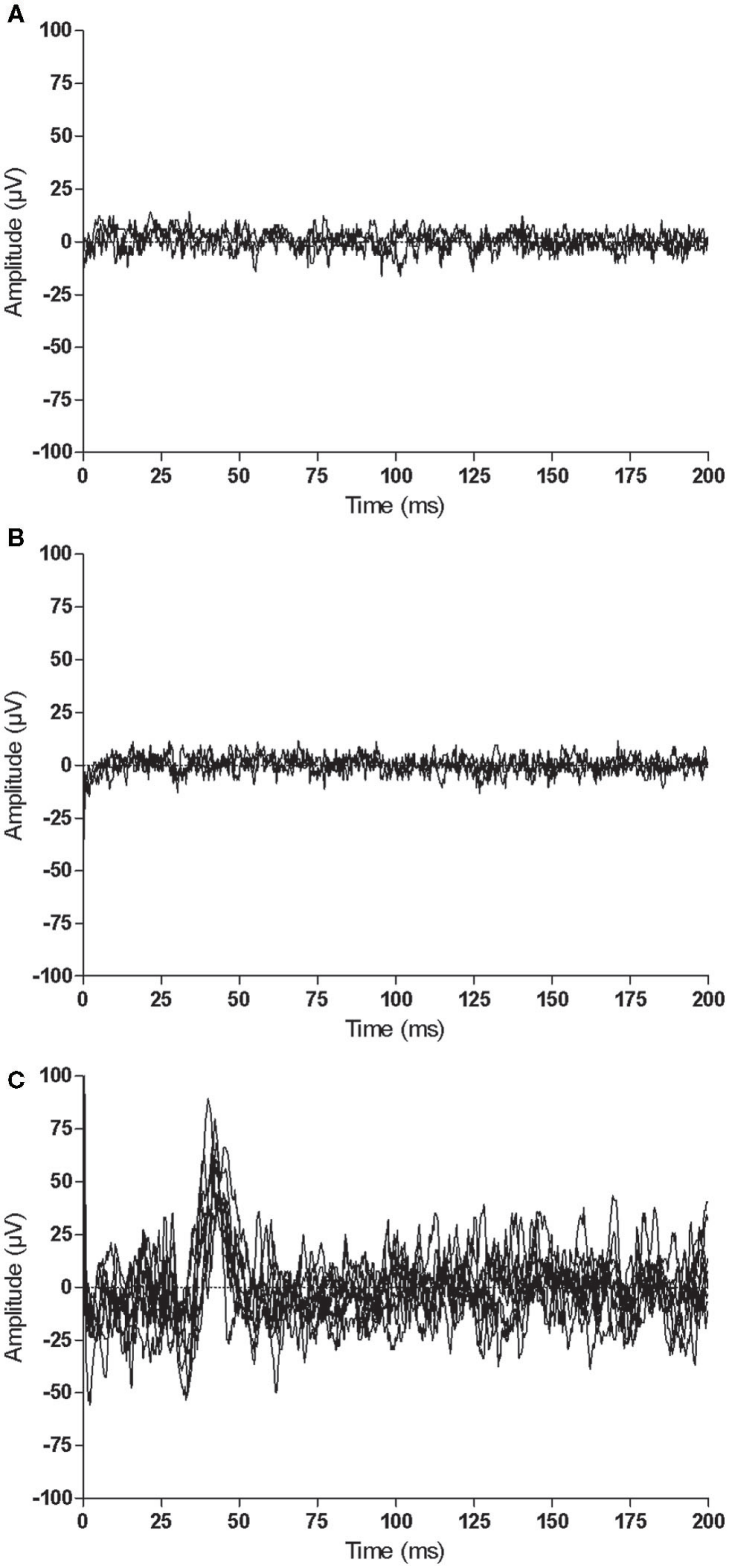
**Representative recordings when stimulating the same cortical territory of the untrained/lesioned M1 for stroke participant S^6^ at each testing session: (A) pre-intervention^1^, (B) pre-intervention^2^, and (C) post-intervention**. MEPs could be elicited between 80 and 100% of maximal stimulator output following the intervention (0 ms corresponds to stimulus delivery).

Similar to the trained M1, there were no consistent trends observed in the untrained M1 recruitment curve or CSP duration for control participants (Figure 7). Although MEPs could be obtained from the paretic EDC for stroke participant S^5^, changes in recruitment curve slope in the untrained/lesioned M1 were not evident from pre-intervention testing (pre-intervention^1^: 0.05%, pre-intervention^2^: 0.01%) to post-intervention testing (0.01%). The same trend was observed even when a subset of samples was evaluated to more precisely match background muscle activity across sessions. MEPs could not be elicited via stimulation of the lesioned M1 for stroke participant S^6^ during the pre-intervention^1^ and pre-intervention^2^ testing sessions, even at maximum stimulator output. Following the intervention, however, MEPs could be readily evoked at a minimum of 80% maximum stimulator output with background muscle activation (Figures 8A–C).

## Discussion

This study examined the effects of high intensity, unilateral resistance training of a non-paretic muscle group on the force-generating capacity of a severely paretic muscle group. Potential neural adaptations underlying an intermanual effect were also explored in a subset of participants. Findings provide evidence that training moderated paresis and enhanced AROM in stroke participants. As anticipated, strength gains were observed in the untrained musculature of control participants. Although only a subset of participants underwent TMS testing, there was also evidence of neurophysiological changes targeting the paretic, untrained musculature in stroke participants.

Gains in the 1-RM of both control and stroke groups’ wrist extensors are similar to previous work that trained the dynamic contraction strength of elbow flexors (25). It might be expected that individuals with neurological deficits cannot engage in training to the same extent as neurologically intact individuals of similar age, even when the training involves the non-paretic limb. Results of the current study suggest otherwise. In fact, there was no difference in training load or percent gain in the trained wrist extensors between stroke and control groups. Although sex was not controlled for between groups, dynamic contraction strength was actually greater in the stroke group at all testing sessions. There was a strong relationship between training load and the relative strength gain in the trained musculature as well. As mentioned previously, some participants in both groups could not achieve the minimum volume at the prescribed intensity (i.e., 80% 1-RM) during initial sessions, but all participants trained well above this minimum intensity by the final training session. Despite the lack of a relationship between training load and the strength gain in the untrained wrist extensors of the control group, there was a strong relationship in the stroke group. Overall, these findings suggest that stroke participants tolerated the training well. Thus, it may be possible to increase training volume within individual sessions beyond the level prescribed in this study, potentially enhancing the resulting strength gain in the paretic, untrained musculature.

In the control group, the relative strength gain in the untrained musculature was approximately half of the gain in the trained musculature, which is consistent with previous work (25, 26, 28). A direct comparison of the relative gain in the stroke group is not possible because AROM was used as a surrogate measure of strength. Nevertheless, there was a strong relationship between percent gains in AROM of the untrained wrist extensors and 1-RM of the trained wrist extensors. To date, only one study has examined the cross education phenomenon in stroke survivors and found that 6 weeks of maximal isometric resistance training increased torque production (31%) and activation (20%) of the untrained, paretic ankle dorsiflexors (11). Results of the current study taken together with those of this previous study suggest that unilateral resistance training enhances force-generating capacity irrespective of contraction type (i.e., dynamic or isometric) or muscle group (i.e., upper or lower extremity).

The primary clinical implication of these findings is that when motor retraining of the affected limb is compromised due to paretic severity, high-intensity resistance training of the non-paretic musculature may be an alternative strategy. Relative to current methods used to manage these cases in clinical practice (e.g., electrical stimulation, passive stretch, etc.), this strategy may deliver a more optimal stimulus to restore movement production. To this end, it might be argued that maximal contractions of the paretic musculature could bring about similar changes in strength. Future research should investigate whether the intervention examined here produces effects that are different from the abovementioned methods currently used in clinical practice and/or maximal contractions of a paretic muscle group.

Consistent with the observed strength gains, the two stroke participants tested for neural adaptations exhibited greater voluntary muscle activation in the untrained, paretic wrist extensors after training. In one stroke participant, MEPs from the untrained/lesioned M1 could not be obtained prior to the intervention but were readily elicited thereafter, suggesting that the intervention enhanced corticospinal excitability. Because of the considerable physiological complexity between the site of stimulation at the level of M1 and recording at the muscle, coupled with the potential for adaptations to occur at one or multiple levels of the neuraxis, the observed change cannot be attributed exclusively to adaptation at the cortical level. Enhanced voluntary muscle activation of the other stroke participant in absence of changes in corticospinal excitability also implies that adaptations are not constrained to corticomotor regions. Changes at the level of the spinal cord and brainstem were not evaluated. Since neurological damage in participant S^6^ was above these levels (Table 2), it can be concluded that net excitation of the corticospinal pathway, inclusive of all inhibitory and excitatory inputs, was increased resulting in enhanced communication between corticomotor and spinal motor neurons. Acknowledging that only a subset of participants in the small sample of participants was tested, additional work on the precise mechanisms involved is warranted.

Contrary to previous work (28), there were no changes in interhemispheric inhibition in the subset of control participants tested. In addition, there were no changes in intracortical inhibition (24, 26) or corticospinal excitability (24, 25). Differences in testing and training protocols as well as the muscle group trained may account for this inconsistency. The most recent of these studies (28), for instance, trained an intrinsic hand muscle (i.e., first dorsal interosseous) with isometric contractions; whereas, an extrinsic hand muscle was trained with dynamic contractions in the current study. In addition, the previous study used a paired-pulse paradigm to evaluate interhemispheric inhibition, while the ISP was used here. Another difference in testing was the timing of the probe relative to the intervention. Interhemispheric inhibition was evaluated immediately following training in the previous study but was examined several days after the final training session in the current study. Whether the resulting neural adaptations outlast training on a short-term (e.g., minutes/hours) or long-term basis (e.g., days) is another consideration for future research to explore. It is worth noting that several studies have examined neural adaptations in response to resistance training, irrespective of the cross education phenomenon, and findings are also quite mixed (4). The current study, however, is limited by the small number of participants in which neural adaptations were explored, so further work is needed.

In stroke participants, ISPs were absent prior to the intervention but suppression in the EMG was more apparent after. One possible explanation may be that the training enhanced accessibility of the circuits mediating the ISP. Alternatively, enhanced muscle activation resulting from the intervention made the suppression more visually discernable. There is limited research available on interhemispheric inhibition post stroke as measured by the ISP. A recent study examined longitudinal changes and found it to be associated with more severe impairment at the onset of rehabilitation but not at later time points (62). Another recent study found short-term reductions in the ISP following repetitive reaching movements (51). In addition, several different definitions have been used to establish criteria for calculating the ISP, each of which have been reported to result in different latencies, durations, and amplitudes (63), complicating a straightforward comparison of results between studies. Thus, it is unclear what type of long-term changes may result from resistance training in persons with stroke.

There are three important limitations of this study that should be taken into consideration. First, despite being able to detect the hypothesized effect, sample sizes for both groups were small. Likewise, only a subset of participants from each sample had viable data from TMS testing, which limited an understanding of potential neural adaptations. Thus, the findings reported here are exploratory in nature. Second, the TMS protocol was limited to single-pulse measures. These measures were selected on the basis of feasibility in a subset of the patient population where cortical physiology is particularly challenging to study (51). Nevertheless, it is possible that corticospinal adaptations may have been detected if different stimulation parameters and/or techniques had been used. Although cervicomedullary or peripheral nerve stimulation techniques were not used, recent work suggests that the neural mechanisms underlying the cross education phenomenon do not reside at subcortical (64) or spinal (65) levels in neurologically intact adults. This does not preclude that possibility that such mechanisms may be involved in individuals with severe neurological deficits. Another limitation of the current study was the indirect measure used to evaluate force production in stroke participants with severely compromised force-generating capacity. AROM was used as a surrogate measure because it could be quantified across time points and, therefore, capture the magnitude of change. Paresis was generally less severe in the sample of the previous study that examined cross education in stroke (11). Four participants in that study, however, could not dorsiflex the ankle to produce a torque prior to training but were able to do so after. Thus, a direct measure of joint kinetics could have supplemented the interpretation of gains from pre- to post-intervention.

## Conclusion

Findings of the current study indicate that high-intensity, unilateral resistance training of a non-paretic muscle group can enhance movement production in a homologous muscle group that is severely impaired. Neurological adaptations that underlie these changes need to be further explored. These findings are clinically relevant, particularly in cases where paretic severity precludes an individual from participating in evidence-based motor retraining programs following stroke. Based on the results reported here, future work is needed to identify the optimal timing and dose of this type of training following stroke. The effects of training different contraction types and muscle group combinations should also be examined. It is also possible that this type of training can be delivered in combination with other adjuvant therapies, such as non-invasive brain stimulation, to further increase force-generating capacity and movement production in individuals with severe post-stroke hemiparesis (66).

## Conflict of Interest Statement

The authors declare that the research was conducted in the absence of any commercial or financial relationships that could be construed as a potential conflict of interest.

## References

[1] Gandevia SC Allen GM Butler JE Taylor JL. Supraspinal factors in human muscle fatigue: evidence for suboptimal output from the motor cortex. J Physiol (1996) 490:529–36. 10.1113/jphysiol.1996.sp021164 8821149 PMC1158689

[2] Gandevia SC. Neural control in human muscle fatigue: changes in muscle afferents, moto neurones and moto cortical drive. Acta Physiol Scand (1998) 162:275–83. 10.1046/j.1365-201X.1998.0299f.x 9578373

[3] Gandevia SC. Spinal and supraspinal factors in human muscle fatigue. Physiol Rev (2001) 81:1725–89. 11581501 10.1152/physrev.2001.81.4.1725

[4] Carroll TJ Selvanayagam VS Riek S Semmler JG. Neural adaptations to strength training: moving beyond transcranial magnetic stimulation and reflex studies. Acta Physiol (2011) 202:119–40. 10.1111/j.1748-1716.2011.02271.x 21382178

[5] Hortobagyi T Lambert NJ Hill JP. Greater cross education following training with muscle lengthening than shortening. Med Sci Sports Exerc (1997) 29:107–12. 10.1097/00005768-199701000-00015 9000162

[6] Munn J Herbert RD Hancock MJ Gandevia SC. Training with unilateral resistance exercise increases contralateral strength. J Appl Physiol (2005) 99:1880–4. 10.1152/japplphysiol.00559.2005 16024518

[7] Scripture E Smith TL Brown EM On the education of muscular control and power. Stud Yale Psychol Lab (1894) 2:114–9.

[8] Shima N Ishida K Katayama K Morotome Y Sato Y Miyamura M. Cross education of muscular strength during unilateral resistance training and detraining. Eur J Appl Physiol (2002) 86:287–94. 10.1007/s00421-001-0559-z 11990741

[9] Yasuda Y Miyamura M Cross transfer effects of muscular training on blood-flow in the ipsilateral and contralateral forearms. Eur J Appl Physiol Occup Physiol (1983) 51:321–9. 10.1007/BF00429068 6685030

[10] Zhou S. Chronic neural adaptations to unilateral exercise: mechanisms of cross education. Exerc Sport Sci Rev (2000) 28:177–84. 11064852

[11] Dragert K Zehr EP. High-intensity unilateral dorsiflexor resistance training results in bilateral neuromuscular plasticity after stroke. Exp Brain Res (2013) 225:93–104. 10.1007/s00221-012-3351-x 23196803

[12] Farthing JP Zehr EP. Restoring symmetry: clinical applications of cross-education. Exerc Sport Sci Rev (2014) 42:70–5. 10.1249/JES.0000000000000009 24508737

[13] Howatson G Zult T Farthing JP Zijdewind I Hortobagyi T. Mirror training to augment cross-education during resistance training: a hypothesis. Front Hum Neurosci (2013) 7:396. 10.3389/fnhum.2013.00396 23898251 PMC3721498

[14] Barker WH Mullooly JP. Stroke in a defined elderly population, 1967-1985 – a less lethal and disabling but no less common disease. Stroke (1997) 28:284–90. 10.1161/01.STR.28.2.284 9040676

[15] Lai SM Studenski S Duncan PW Perera S. Persisting consequences of stroke measured by the stroke impact scale. Stroke (2002) 33:1840–4. 10.1161/01.STR.0000019289.15440.F2 12105363

[16] Kolominsky-Rabas PL Weber M Gefeller O Neundoerfer B Heuschmann PU. Epidemiology of ischemic stroke subtypes according to TOAST criteria: incidence, recurrence, and long-term survival in ischemic stroke subtypes: a population-based study. Stroke (2001) 32:2735–40. 10.1161/hs1201.100209 11739965

[17] Murray CJL Lopez AD. Mortality by cause for eight regions of the world: global burden of disease study. Lancet (1997) 349:1269–76. 10.1016/S0140-6736(96)07493-4 9142060

[18] Thrane G Friborg O Anke A Indredayik B. A meta-analysis of constraint-induced movement therapy after stroke. J Rehabil Med (2014) 46:833–42. 10.2340/16501977-1859 25182341

[19] Veerbeek JM van Wegen E van Peppen R van der Wees PJ Hendriks E Rietberg M What is the evidence for physical therapy poststroke? A systematic review and meta-analysis. PLoS One (2014) 9:e87987. 10.1371/journal.pone.0087987 24505342 PMC3913786

[20] Rossini PM Calautti C Pauri F Baron JC. Post-stroke plastic reorganisation in the adult brain. Lancet Neurol (2003) 2:493–502. 10.1016/S1474-4422(03)00485-X 12878437

[21] Sathian K Buxbaum LJ Cohen LG Krakauer JW Lang CE Corbetta M Neurological principles and rehabilitation of action disorders: common clinical deficits. Neurorehabil Neural Repair (2011) 25:21s–32s. 10.1177/1545968311410941 21613535 PMC4139495

[22] Murase N Duque J Mazzocchio R Cohen LG. Influence of interhemispheric interactions on motor function in chronic stroke. Ann Neurol (2004) 55:400–9. 10.1002/ana.10848 14991818

[23] Duque J Hummel F Celnik P Murase N Mazzocchio R Cohen LG Transcallosal inhibition in chronic subcortical stroke. Neuroimage (2005) 28:940–6. 10.1016/j.neuroimage.2005.06.033 16084737

[24] Goodwill AM Pearce AJ Kidgell DJ Corticomotor plasticity following unilateral strength training. Muscle Nerve (2012) 46:384–93. 10.1002/mus.23316 22907229

[25] Kidgell DJ Stokes MA Pearce AJ. Strength training of one limb increases corticomotor excitability projecting to the contralateral homologous limb. Motor Control (2011) 15:247–66. 21628728 10.1123/mcj.15.2.247

[26] Latella C Kidgell DJ Pearce AJ. Reduction in corticospinal inhibition in the trained and untrained limb following unilateral leg strength training. Eur J Appl Physiol (2012) 112:3097–107. 10.1007/s00421-011-2289-1 22200796

[27] Lee M Gandevia SC Carroll TJ. Unilateral strength training increases voluntary activation of the opposite untrained limb. Neurophysiol Clin (2009) 120:802–8. 10.1016/j.clinph.2009.01.002 19230754

[28] Hortobagyi T Richardson SP Lomarev M Shamim E Meunier S Russman H Interhemispheric plasticity in humans. Med Sci Sports Exerc (2011) 43:1188–99. 10.1249/MSS.0b013e31820a94b8 21200340 PMC4137570

[29] Oldfield RC The assessment and analysis of handedness: the Edinburgh inventory. Neuropsychologia (1971) 9:97–113. 10.1016/0028-3932(71)90067-4 5146491

[30] Dragert K Zehr EP. Bilateral neuromuscular plasticity from unilateral training of the ankle dorsiflexors. Exp Brain Res (2011) 208:217–27. 10.1007/s00221-010-2472-3 21069308

[31] Cortes M Black-Schaffer RM Edwards DJ. Transcranial magnetic stimulation as an investigative tool for motor dysfunction and recovery in stroke: an overview for neurorehabilitation clinicians. Neuromodulation (2012) 15:316–25. 10.1111/j.1525-1403.2012.00459.x 22624621 PMC3760962

[32] Farthing JP Chilibeck PD Binsted G. Cross-education of arm muscular strength is unidirectional in right-handed individuals. Med Sci Sports Exerc (2005) 37:1594–600. 10.1249/01.mss.0000177588.74448.75 16177613

[33] Hakkennes S Keating JL. Constraint-induced movement therapy following stroke: a systematic review of randomised controlled trials. Aust J Physiother (2005) 51:221–31. 10.1016/S0004-9514(05)70003-9 16321129

[34] Munn J Herbert RD Hancock MJ Gandevia SC Resistance training for strength: effect of number of sets and contraction speed. Med Sci Sports Exerc (2005) 37:1622–6. 10.1249/01.mss.0000177583.41245.f8 16177617

[35] Carroll TJ Herbert RD Munn J Lee M Gandevia SC. Contralateral effects of unilateral strength training: evidence and possible mechanisms. J Appl Physiol (2006) 101:1514–22. 10.1152/japplphysiol.00531.2006 17043329

[36] Cernacek J Contralateral motor irradiation – cerebral dominance. Its changes in hemiparesis. Arch Neurol (1961) 4:165–72. 10.1001/archneur.1961.00450080047005 13691977

[37] Chaco J Blank A Mirror movements in hemiparesis. Confin Neurol (1974) 36:1–4. 10.1159/000102779 4824972

[38] Hopf HC Schlegel HJ Lowitzsch K Irradiation of voluntary activity to the contralateral side in movements of normal subjects and patients with central motor disturbances. Eur Neurol (1974) 12:142–7. 10.1159/000114613 4426322

[39] Peterson MD Rhea MR Alvar BA. Applications of the dose-response for muscular strength development: a review of meta-analytic efficacy and reliability for designing training prescription. J Strength Cond Res (2005) 19:950–8. 10.1519/00124278-200511000-00038 16287373

[40] Morrissey MC Harman EA Johnson MJ. Resistance training modes – specificity and effectiveness. Med Sci Sports Exerc (1995) 27:648–60. 10.1249/00005768-199505000-00006 7674868

[41] Beebe JA Lang CE. Active range of motion predicts upper extremity function 3 months after stroke. Stroke (2009) 40:1772–9. 10.1161/STROKEAHA.108.536763 19265051 PMC2718540

[42] Bohannon RW Smith MB. Interrater reliability of a modified Ashworth scale of muscle spasticity. Phys Ther (1987) 67:206–7. 3809245 10.1093/ptj/67.2.206

[43] Lang CE Bland MD Bailey RR Schaefer SY Birkenmeier RL. Assessment of upper extremity impairment, function, and activity after stroke: foundations for clinical decision making. J Hand Ther (2013) 26:104–14. 10.1016/j.jht.2012.06.005 22975740 PMC3524381

[44] Malcolm M Triggs WJ Light KE Shechtman O Khandekar G Gonzalez Rothi LJ. Reliability of motor cortex transcranial magnetic stimulation in four muscle representations. Neurophysiol Clin (2006) 117:1037–46. 10.1016/j.clinph.2006.02.005 16564206

[45] Cicinelli P Traversa R Rossini PM. Post-stroke reorganization of brain motor output to the hand: a 2-4 month follow-up with focal magnetic transcranial stimulation. Electroencephalogr Clin Neurophysiol (1997) 105:438–50. 10.1016/S0924-980X(97)00052-0 9448645

[46] Traversa R Cicinelli P Oliveri M Palmieri MG Filippi MM Pasqualetti P Neurophysiological follow-up of motor cortical output in stroke patients. Neurophysiol Clin (2000) 111:1695–703. 10.1016/S1388-2457(00)00373-4 10964084

[47] Rothwell J Hallett M Berardelli A Eisen A Rossini P Paulus W. Magnetic stimulation: motor evoked potentials. The international federation of clinical neurophysiology. Electroencephalogr Clin Neurophysiol Suppl (1999) 52:97–103. 10590980

[48] Fuhr P Agostino R Hallett M. Spinal motor-neuron excitability during the silent period after cortical stimulation. Electroencephalogr Clin Neurophysiol (1991) 81:257–62. 10.1016/0168-5597(91)90011-L 1714819

[49] Roick H Vongiesen HJ Benecke R. On the origin of the postexcitatory inhibition seen after transcranial magnetic brain-stimulation in awake human-subjects. Exp Brain Res (1993) 94:489–98. 10.1007/BF00230207 8359263

[50] Mccormick DA. Gaba as an inhibitory neurotransmitter in human cerebral-cortex. J Neurophysiol (1989) 62:1018–27. 2573696 10.1152/jn.1989.62.5.1018

[51] Harris-Love ML Morton SM Perez MA Cohen LG. Mechanisms of short-term training-induced reaching improvement in severely hemiparetic stroke patients: a TMS study. Neurorehabil Neural Repair (2011) 25:398–411. 10.1177/1545968310395600 21343522 PMC3098309

[52] Chowdhury SA Matsunami KI. GABA-B-related activity in processing of transcallosal response in cat motor cortex. J Neurosci Res (2002) 68:489–95. 10.1002/jnr.10223 11992476

[53] Rothwell JC Colebatch J Britton TC Priori A Thompson PD Day BL Physiological-studies in a patient with mirror movements and agenesis of the corpus-callosum. J Physiol (1991) 438:34–34.

[54] Meyer BU Roricht S Voneinsiedel HG Kruggel F Weindl A. Inhibitory and excitatory interhemispheric transfers between motor cortical areas in normal humans and patients with abnormalities of the corpus-callosum. Brain (1995) 118:429–40. 10.1093/brain/118.2.429 7735884

[55] Heinen F Glocker FX Fietzek U Meyer BU Lucking CH Korinthenberg R. Absence of transcallosal inhibition following focal magnetic stimulation in preschool children. Ann Neurol (1998) 43:608–12. 10.1002/ana.410430508 9585354

[56] Boroojerdi B Diefenbach K Ferbert A. Transcallosal inhibition in cortical and subcortical cerebral vascular lesions. J Neurol Sci (1996) 144:160–70. 10.1016/S0022-510X(96)00222-5 8994119

[57] Wassermann EM Fuhr P Cohen LG Hallett M. Effects of transcranial magnetic stimulation on ipsilateral muscles. Neurology (1991) 41:1795–9. 10.1212/WNL.41.11.1795 1944911

[58] Wassermann EM Pascualleone A Hallett M Cortical motor representation of the ipsilateral hand and arm. Exp Brain Res (1994) 100:121–32. 10.1007/BF00227284 7813640

[59] Carroll TJ Riek S Carson RG. Reliability of the input-output properties of the cortico-spinal pathway obtained from transcranial magnetic and electrical stimulation. J Neurosci Methods (2001) 112:193–202. 10.1016/S0165-0270(01)00468-X 11716954

[60] Chen R Yung D Li JY. Organization of ipsilateral excitatory and inhibitory pathways in the human motor cortex. J Neurophysiol (2003) 89:1256–64. 10.1152/jn.00950.2002 12611955

[61] Pennisi G Alagona G Bella R Rapisarda G Greco S Nicoletti F Transcranial magnetic stimulation after pure motor stroke. Neurophysiol Clin (2002) 113:1536–43. 10.1016/S1388-2457(02)00255-9 12350429

[62] Takechi U Matsunaga K Nakanishi R Yamanaga H Murayama N Mafune K Longitudinal changes of motor cortical excitability and transcallosal inhibition after subcortical stroke. Neurophysiol Clin (2014) 125:2055–69. 10.1016/j.clinph.2014.01.034 24636830

[63] Nagaya EM Ferreiro de Andrade KN da Rocha Correa CE Conforto AB Impact of different definitions of the ipsilateral silent periods on measurements of interhemispheric inhibition in patients with stroke and healthy subjects. J Neurol Disord Stroke (2014) 2:1–7.

[64] Hortobagyi T Taylor JL Petersen NT Russell G Gandevia SC. Changes in segmental and motor cortical output with contralateral muscle contractions and altered sensory inputs in humans. J Neurophysiol (2003) 90:2451–9. 10.1152/jn.01001.2002 14534271

[65] Lagerquist O Zehr EP Docherty D. Increased spinal reflex excitability is not associated with neural plasticity underlying the cross-education effect. J Appl Physiol (2006) 100:83–90. 10.1152/japplphysiol.00533.2005 16357081

[66] Conforto AB Anjos SM Saposnik G Mello EA Nagaya EM Santos W Transcranial magnetic stimulation in mild to severe hemiparesis early after stroke: a proof of principle and novel approach to improve motor function. J Neurol (2012) 259:1399–405. 10.1007/s00415-011-6364-7 22173953 PMC4883097

